# Molecular engineering of several butterfly-shaped hole transport materials containing dibenzo[*b,d*]thiophene core for perovskite photovoltaics

**DOI:** 10.1038/s41598-022-18469-1

**Published:** 2022-08-17

**Authors:** Zahra Shariatinia, Seyed-Iman Sarmalek

**Affiliations:** Department of Chemistry, Amirkabir University of Technology (Tehran Polytechnic), P.O.Box:15875-4413, Tehran, Iran

**Keywords:** Chemistry, Energy

## Abstract

Several butterfly-shaped materials composed of dibenzo[b,d]thiophene (DBT) and dibenzo-dithiophene (DBT5) cores were designed as hole transporting materials (HTMs) and their properties were studied by density functional theory (DFT) computations for usage in mesoscopic n-i-p perovskite solar cells (PSCs). To choose suitable HTMs, it was displayed that both of lowest unoccupied molecular orbital (LUMO) and highest occupied molecular orbital (HOMO) energies of molecules were located higher than those of CH_3_NH_3_PbI_3_ (MAPbI_3_) perovskite as they were able to transfer holes from the MAPbI_3_ toward Ag cathode. Negative solvation energy (ΔE_solvation_) values for all HTMs (within the range of − 5.185 to − 18.140 kcal/mol) revealed their high solubility and stability within CH_2_Cl_2_ solvent. The DBT5-COMe demonstrated the lowest values of band gap (E_g_ = 3.544) and hardness (η = 1.772 eV) (the greatest chemical activity) and DBT5-CF_3_ displayed the biggest η = 1.953 eV (maximum stability) that were predominantly valuable for effective HTMs. All HTMs presented appropriately high LHEs from 0.8793 to 0.9406. In addition, the DBT5 and DBT5-SH depicted the lowest exciton binding energy (E_b_) values of 0.881 and 0.880 eV which confirmed they could produce satisfactory results for the PSCs assembled using these materials. The DBT5-SH and DBT5-H had maximum hole mobility (μ_h_) values of 6.031 × 10^–2^ and 1.140 × 10^–2^ which were greater than those measured for the reference DBT5 molecule (μ_h_ = 3.984 × 10^–4^ cm^2^/V/s) and about 10 and 100 times superior to the calculated and experimental μ_h_ values for well-known Spiro-OMeTAD. The DBT5-COOH illustrated the biggest open circuit voltage (V_OC_), fill factor (FF) and power conversion efficiency (PCE) values of 1.166 eV, 0.896 and 23.707%, respectively, establishing it could be as the best HTM candidate for high performance PSCs.

## Introduction

Global energy consumption is predicted to be doubled by 2050, owing to fast population and economic expansion^1,2^. On the other hand, adverse effects of increasing global warming on mankind and the universal biosphere necessitate immediate emission reductions^3–5^. As a result, it is unavoidable that fossil fuels to be replaced by ecologically beneficial and renewable fuels^6,7^. Regarding the huge amount of sunlight that reaches the planet, it is known as one of the most important renewable energy sources, prompting a worldwide effort to develop photovoltaic energy conversion technology^8–10^.

The demand for high-efficiency, low-cost photovoltaic systems is continually driving the development of new solar cells ahead^11,12^. Among various types of solar cells, PSCs have attracted a lot of interest because of their excellent advantages of long length of charge carriers’ diffusions, great absorption coefficients plus simple manufacturing techniques^13,14^, so that their PCE has rapidly boosted beyond 25% in few years^15–17^. PSCs are currently the most likely solar cells to be commercialized in the near future similar to the silicon solar cells^18,19^. Massive optimization strategies have been developed to improve the PSC devices’ efficiency and stability^20,21^. For this purpose, HTMs have been investigated as highly beneficial components in PSCs to improve device performance^22,23^.

HTMs are one of the most essential components of PSCs that has an impact on their performance^24,25^. The discovery of HTMs is critical since these materials are critical for charge extraction, and PSC stability^26,27^. The PCEs of PSCs boosted rapidly utilizing HTMs, particularly the well-known 2,2′,7,7′-tetrakis(*N*,*N*-di-*p*-methoxyphenylamine)-9,9′-spirobifluorene (Spiro-OMeTAD)^28,29^. Nonetheless, Spiro-OMeTAD has several drawbacks as it shows low stability, high cost and must be used along with two expensive dopants to afford favorable PCEs^30^. Therefore, researchers are trying to achieve various effective HTMs of lower costs to substitute Spiro-OMeTAD^31^. In this context, HTMs with D-A-D (donor–acceptor–donor) backbones have exhibited appropriate PCEs for PSCs because of their suitable energy levels plus charge transmission characteristics^31–33^.

The dibenzothiophene (DBT) derivatives have been used as HTMs in PSCs and other opto-electronic devices and showed favorable results. For instance, Zhang et al. synthesized a HTM with DBT core and obtained a high PCE of 21.12% for planar PSCs with 83.25% fill factor confirming promising influence of this molecule which makes it a viable HTM candidate for planar p-i-n PSCs^34^. In another work, the PSCs fabricated using HTMs based on dibenzoquinquethiophene and dibenzosexithiophene revealed PCEs of 18.1 and 14.3%, respectively^35^. Recently, the PSCs assembled with two dopant-free HTMs including a DBT-core containing molecule and pure Spiro-OMeTAD demonstrated PCEs of 13.61 and 9.34% which approved the DBT-based HTM has a higher performance than the pure Spiro-OMeTAD^36^. Another group synthesized three DBT-based HTMs for PSCs and displayed that the PCEs of devices containing these HTMs were 8.99, 10.67 and 20.90% which were greater than the PCE = 4.69% for the device with the pure Spiro-OMeTAD^37^. Similarly, it was exhibited that when central pyrrole ring in dithienopyrrole is substituted by benzene ring to create benzodithiophene, the PCE enhances from 15.6 to 18.1%^38^.

The electronic, optical and structural properties of HTMs employing in PSCs can be explored through DFT studies^39^. So far, lots of computational efforts have been performed to connect opto-electronic features of HTMs with their structural and electronic properties^40^. In fact, the molecular structures of HTM can significantly change its hole extraction capacity from perovskite^41^. Consequently, numerous HTMs are designed and studied in order to achieve appropriate compounds indicating high hole mobility and stability^42,43^ which are easily synthesized by low cost methods and extremely boost PSCs’ stability and efficiency^44^.

**Figure 1:**
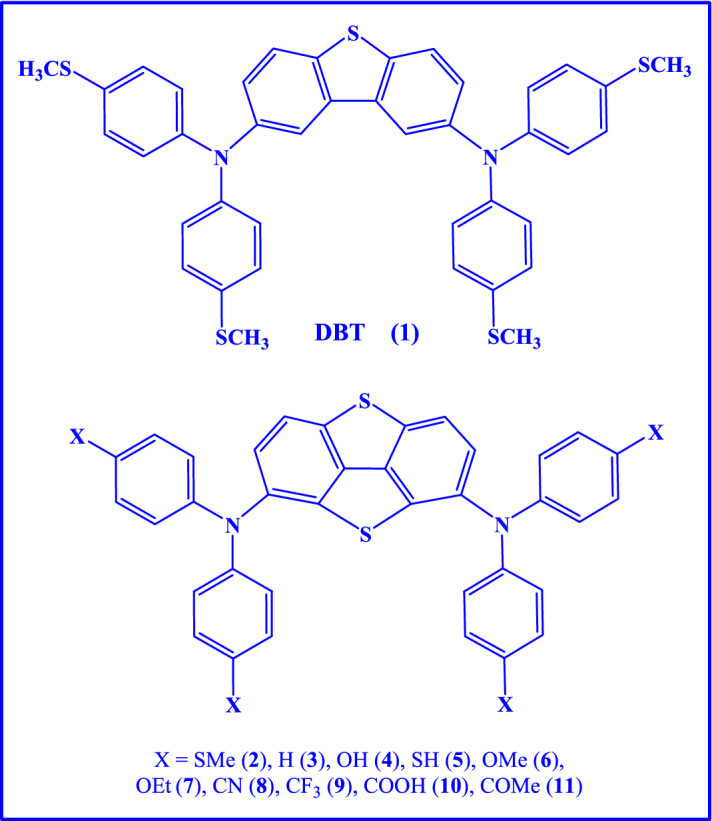
The molecular structures of the designed HTMs **1–11**.

As stated above, the planar p-i-n PSCs composed of the DBT HTM indicated a high PCE = 21.12% and fill factor of 83.25%^34^. Hence, in this study, we decided to use this HTM in mesoscopic n-i-p PSCs in order to find whether it is also suitable for mesoscopic n-i-p devices. For this purpose, some butterfly-shaped HTMs were designed by modifying the DBT core via attachment of another thiophene ring to the central part of DBT and changing X substituents onto *para*-positions of *N,N*-diphenylamine moieties, where X = SMe, H, OH, SH, OMe, OEt, CN, CF_3_, COOH and COMe (see Fig. 1). DFT computations were carried out to investigate hole mobility, structures, plus electronic and optical features of DBT-based HTMs for application in PSCs. Results verified that the DBT5-SH and DBT5-H samples showed the greatest hole mobilities (μ_h_ = 6.031 × 10^–2^ and 1.140 × 10^–2^ cm^2^/V/s) which were beyond those of DBT and DBT5 molecules as well as Spiro-OMeTAD (calculated μ_h_ = 5.65 × 10^–3^ cm^2^/V/s, experimental μ_h_ = 4.53 × 10^–4^ cm^2^/V/s). Additionally, the DBT5-COOH illustrated the biggest V_OC_, FF and PCE values of 1.166 eV, 0.896 and 23.707%, respectively. Therefore, this material was suggested as a highly efficient candidate for PSCs fabrication which could possibly lead to higher PCEs than that measured using the pure DBT.

## Methods of computations

The DFT geometry optimization computations were carried out in CH_2_Cl_2_ solution on several butterfly-shaped HTMs **1–11** at B3LYP-D3/6-31G(d,p) method by Gaussian 09 software^45^. The conductor-like polarizable continuum model (C-PCM) method was adopted to estimate solvent influence^46^. It is noteworthy that B3LYP-D3/6-31G(d,p) method was employed herein as it is commonly utilized by many researchers and known as a method of high validity affording appreciated data in real-time and this makes it a reliable and economical method. As an example, electrochemical features of HTMs for solar devices were predicted using the DFT computations at B3LYP-D3/6-31G(d,p) level^47–49^.

The infrared (IR) spectra were obtained by the freq keyword in DFT calculations until no imaginary (negative) frequencies were obtained. Results of frequency calculations on HTMs **1–11** depicted absence of imaginary frequencies. The UV–Visible (UV–Vis) and photoluminescence (PL) spectra were attained through time-dependent density functional theory (TD-DFT) calculations at the B3LYP-D3/6-31G(d,p) method in which number of states was 30, i.e. TD = (nstates = 30). GaussView 5 software was used to obtain the contour and surface maps, IR, UV–Vis and PL spectra, and molecular orbitals^50^.

Inside an organic π-conjugated molecule, the charge transference occurs by non-coherent hopping mechanism due to electron–phonon coupling is noticeably stronger than electronic coupling under ambient conditions. According to Marcus theory, Eq. (1) is used to estimate the hole hopping rate (k_h_), where h, k_B_, T, V and λ, respectively, reveal Planck’s constant, Boltzmann constant, absolute temperature, transfer integral and reorganization energy^51^.

1 $${k}_{h}=\frac{{4\uppi }^{2}}{h}{V}^{2}\frac{1}{\sqrt{4\pi \lambda {k}_{B}T}}exp\left[-\frac{\lambda }{4{k}_{B}T}\right].$$

The λ value can be achieved using Eq. (2), where ${E}_{0}$ and ${E}_{+}$, respectively, signify energies of optimized neutral and cationic molecules whereas the ${E}_{0}^{*}$ and ${E}_{+}^{*}$ respectively show energies of neutral and cationic samples at cationic and neutral states^52^.

2 $$\lambda ={(E}_{+}^{*}-{E}_{+}) {+ (E}_{0}^{*}-{E}_{0}).$$

The transfer integral (V) is computed by Eq. (3), in which e_ii_, e_jj_ exhibit site energies of $\left\langle {\psi_{i} |\hat{H}|\psi_{i} } \right\rangle$, $\left\langle {\psi_{j} |\hat{H}|\psi_{j} } \right\rangle$ while S_ij_ and J_ij_ denote $\left\langle {\psi_{i} |\psi_{j} } \right\rangle$ overlap matrix element and $\left\langle {\psi_{i} |\hat{H}|\psi_{j} } \right\rangle$ transfer integral, respectively^53,54^.

3 $$V= \frac{{J}_{ij}-0.5{S}_{ij}({e}_{ii}+{e}_{jj})}{1-{S}_{ij}^{2}}.$$

The hole mobility (μ_h_) is calculated by Eq. (4), where D and e indicate diffusion coefficient and electron charge, respectively. The D is obtained by Eq. (5), where r_m_, d and m show centroid to centroid distance, spatial dimension, specific hopping route, ${p}_{m}={k}_{m}/\sum_{m}{k}_{m}$ displays probability of hopping process^55^.

4 $$\mu =\frac{e}{{K}_{B}T}D,$$

5 $$D=\underset{t\to \infty }{\mathrm{lim}}\frac{1}{2d}\frac{\langle {x(t)}^{2}\rangle }{t}\approx \frac{1}{2d}\sum_{m}{r}_{m}^{2}{k}_{m}{p}_{m}.$$

The adiabatic ionization potential (IP) was measured by IP = E_+_ − E_0_ equation, where E_0_ and E_+_ illustrated, respectively, energies of neutral and cationic optimized molecules^56^.

The crystalline structures of HTMs **1–11** were simulated by means of polymorph module within Material Studio software^57^ through computing ten most important space groups of Cc, C2, P-1, C2/c, Pbcn, P2_1_, P2_1_/c, Pbca, P2_1_2_1_2_1_, Pna2_1_. To predict all crystal structures and unit cells, the optimized structure is firstly entered into the Materials Studio and then its ESP charges are computed using DMol3 module/calculation (Task: Energy, Basis set: DNP, Properties: Electrostatics and population analysis are selected). Then, the total charges are set to zero using DMol3 module/Analysis/Population analysis. Secondly, all crystal structures and 10 unit cells are predicted by Polymorph module/calculation with choosing the parameters as force field: Dreiding, charges: use current (ESP), quality: Fine, electrostatic: Ewald, van der Waals: Ewald and Space Groups: thick all items. Finally, the Polymorph module/Analysis is selected and all of the predicted crystal structures and unit cells are listed which will be sorted based on their total energies and the most stable crystal structure (of minimum total energy) is selected.

## Results and discussion

### Binding energy, polarizability and solubility

**Figure 2:**
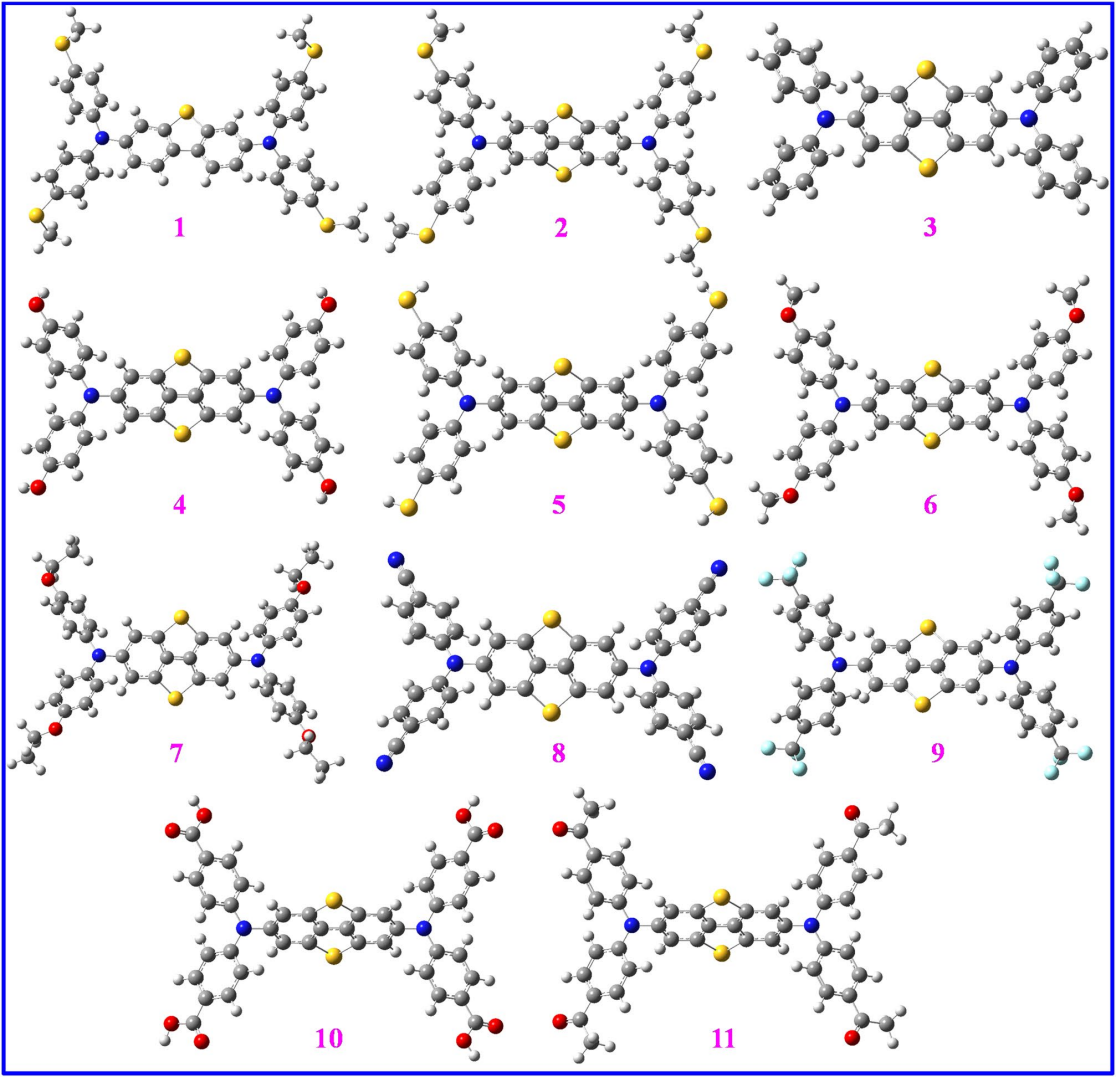
The optimized structures of compounds **1–11** computed at B3LYP-D3/6-31G(d,p) level. This figure was created by Gauss View 5 software which can be found at: https://gaussview.software.informer.com/5.0/.

In this work, several butterfly-shaped HTMs were designed for mesoscopic n-i-p PSCs via addition of another thiophene ring onto the DBT core so that 11 molecules were obtained in which different substituents were placed onto the diphenylamine groups. The structures HTMs **1–11** (Fig. 1) were optimized using B3LYP-D3/6-31G(d,p). Moreover, Fig. 2 exhibits that diverse substituents located onto the *para*-positions of phenyl moieties influence spatial arrangements of these molecules. Therefore, to evaluate the HTMs stability, the binding energy (ΔE_binding_) values are attained. Table 1 shows the ΔE_binding_, ΔE_solvation_, dipole moments and polarizabilities of HTMs **1–11**. As well, ΔE_binding_ stands for the energy amount released upon a molecule formation so that a greater ΔE_binding_ reveals its superior stability. An appropriate HTM shows both high stability and solubility within solution. It is obvious that different ΔE_binding_ values are measured by changing the substituents so that the DBT5-H and DBT5-OEt, respectively, demonstrate the smallest and the utmost energies (− 7780.763 and − 10,536.068 kcal/mol).

**Table 1:** The ΔE_binding_ (binding energy), solvation energy (ΔE_solvation_), dipole moment and polarizability of HTMs **1–11** computed at B3LYP-D3/6-31G(d,p) level.

No.	Label	Formula	ΔE_binding_ (kcal/mole)	ΔE_solvation_ (kcal/mole)	Dipole moment (Debye)	Polarizability (Bohr^3^)
1	DBT	C_40_H_34_N_2_S_5_	− 9270.760	− 10.475	3.9835	817.05
2	DBT5	C_40_H_32_N_2_S_6_	− 9177.172	− 10.350	3.6072	841.33
3	DBT5-H	C_36_H_24_N_2_S_2_	− 7780.763	− 5.185	0.0030	633.58
4	DBT5-OH	C_36_H_24_N_2_O_4_S_2_	− 8188.799	− 13.460	3.2866	670.18
5	DBT5-SH	C_36_H_24_N_2_S_6_	− 7993.744	− 10.340	2.3350	767.13
6	DBT5-OMe	C_40_H_32_N_2_O_4_S_2_	− 9339.964	− 8.999	3.0806	738.68
7	DBT5-OEt	C_44_H_40_N_2_O_4_S_2_	− 10,536.068	− 8.694	2.7496	798.80
8	DBT5-CN	C_40_H_20_N_6_S_2_	− 8579.084	− 18.140	0.0028	760.08
9	DBT5-CF_3_	C_40_H_20_F_12_N_2_S_2_	− 9155.772	− 7.274	0.0017	693.54
10	DBT5-COOH	C_40_H_24_N_2_O_8_S_2_	− 9332.012	− 15.929	3.5028	770.14
11	DBT5-COMe	C_44_H_32_N_2_O_4_S_2_	− 10,053.990	− 14.756	8.5837	825.86

CH_3_ = Me, Et = CH_2_CH_3_.

The HTMs solvation energies were achieved using the formula ΔE_solvation_ = ΔE_solution_ − ΔE_gas_, where ΔE_gas_ and ΔE_solution_, respectively, show the binding energies within gaseous and solution states. All ΔE_solvation_ values in Table 1 are negative confirming high solubility of all HTMs in CH_2_Cl_2_ solvent. Notably, an efficient HTM must be essentially extremely soluble to obtain a uniform layer onto the perovskite film which thereby assists hole transfer at interface of HTM/perovskite. The lowest and highest ΔE_solvation_ values of molecules **1–11** vary from − 5.185 to − 18.140 kcal/mol for the DBT5-H and DBT5-CN, respectively. Also, the high ΔE_solvation_ values of − 18.140, − 15.929, − 14.756 and − 13.460 kcal/mol for the DBT5-CN, DBT5-COOH, DBT5-COMe and DBT5-OH HTMs can be correlated to their high capacities of strong hydrogen bonds formation with solvent molecules. On the other hand, HTMs with lower ΔE_solvation_ values form medium or weak hydrogen bonds with CH_2_Cl_2_ solvent.

Comparing dipole moments of the HTMs **1–11** displays that the DBT5-COMe molecule with has the utmost dipole moment of 8.5837 D but three DBT5-H, DBT5-CN and DBT5-CF_3_ HTMs indicate the smallest dipole moments of 0.0030, 0.0028 and 0.0017 D, respectively. These results can be attributed to the symmetric or asymmetric distributions of positive and negative charges over the structures which lead to lowest and highest dipole moments. Also, it is observed that the dipole moment of DBT (3.9835 D) is larger than those of other DBT5-based molecules except for the DBT5-COMe.

Polarizability of a material corresponds to the electron polarization energy^58^. Hence, a molecule with a larger polarizability should exhibit a more negative electron polarization energy. Among samples **1–11**, the DBT5 and DBT5-H exhibit the greatest and minimum polarizability values of 841.33 and 633.58 Bohr^3^, respectively. As well, comparing the polarizability values of DBT5-H, DBT5-OH and DBT5-SH, respectively (633.58, 670.18 and 767.13 Bohr^3^) confirms that the polarizability enhances by the molecular volume. Similarly, the DBT5-OMe, DBT5-OEt and DBT5-COOH, DBT5-COMe illustrate polarizability values of 738.68, 798.80 and 770.14, 841.33 Bohr^3^, respectively. In fact, a HTM compound with a greater polarizability is advantageous as it may show a smaller electron polarization energy which leads to a simpler formation of charged species and facilitates the charge transfer from the MAPbI_3_ perovskite to the cathode electrode in PSCs.

### Contours and surfaces

**Figure 3:**
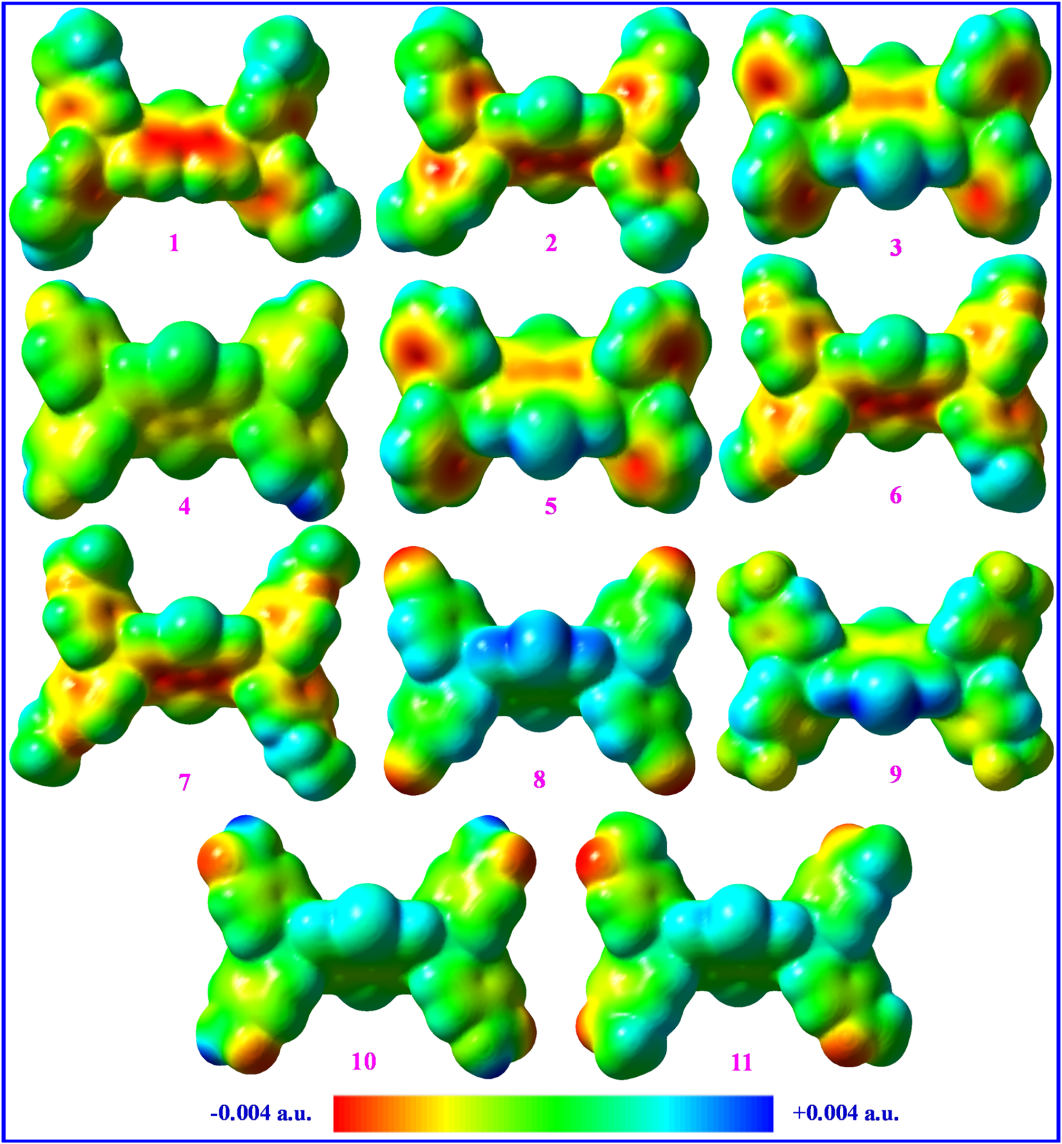
The electrostatic surface potentials of HTMs **1–11** (positive and negative charges are shown by yellow and red colors, respectively). This figure was created by Gauss View 5 software which can be found at: https://gaussview.software.informer.com/5.0/.

Figure 3 presents electrostatic surface potentials (ESPs) for compounds **1–11** that shows charge distributions onto the surfaces of such butterfly-shaped structures. The ESPs demonstrate electrical charge distributions which can be a measure of molecular polarity so that a more symmetric ESP exhibits that the material has a smaller dipole moment. Furthermore, the ESP may be accounted for the structural stability as a more symmetric charge dispersion may reveal that weaker inter-/intra-molecular interactions occur and the molecule is less reactive. Thus, the DBT5-COMe indicating the most asymmetric charge distribution has the utmost reactivity but the DBT5-H, DBT5-CN and DBT5-CF_3_ samples with the most symmetric charge distributions are the most stable structures. Such results are in consistent with the contour maps achieved for these materials.

**Figure 4:**
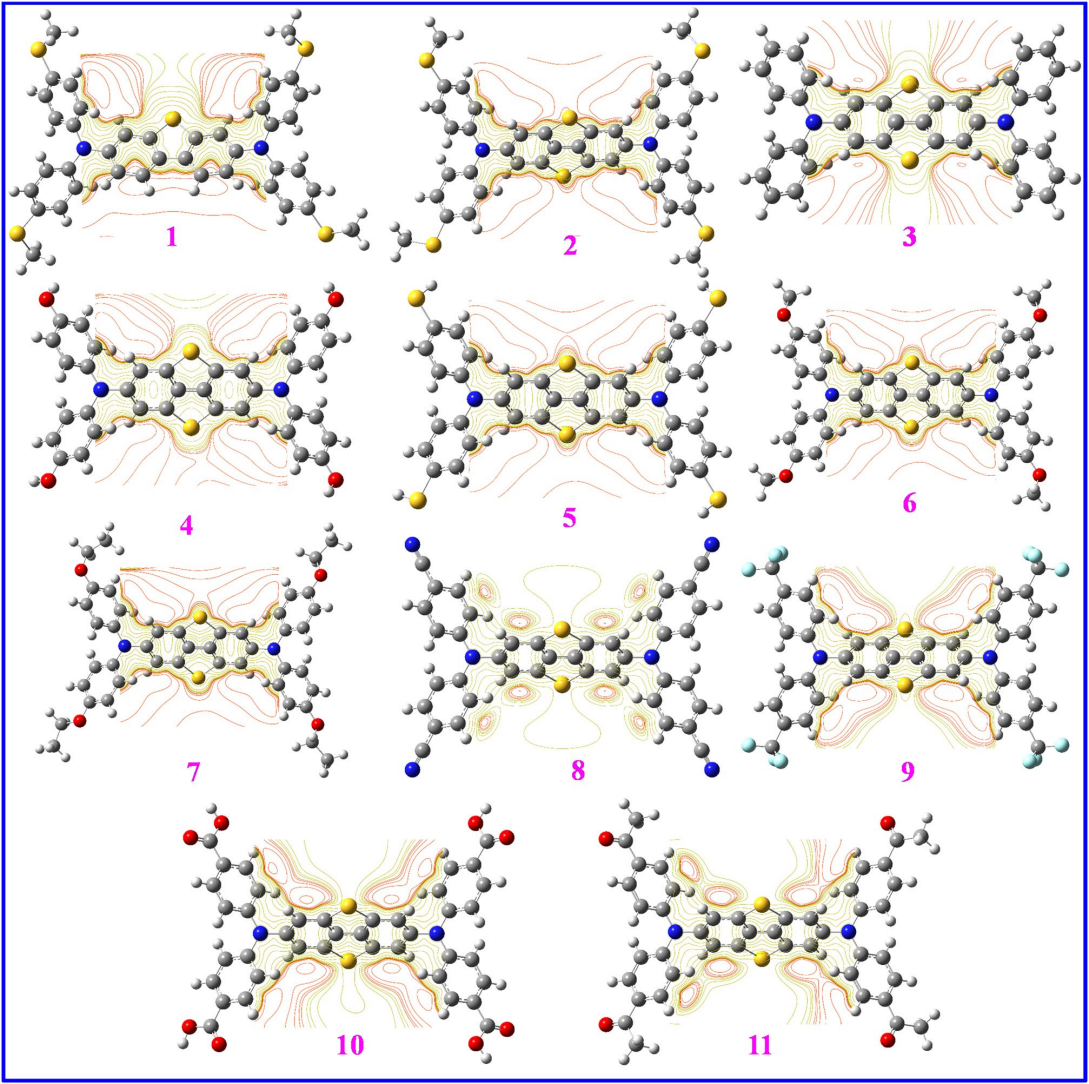
The contour maps for HTMs **1–11** computed at B3LYP-D3/6-31G(d,p) level (red and yellow colors represent negative and positive regions of the wave functions, respectively). This figure was created by Gauss View 5 software which can be found at: https://gaussview.software.informer.com/5.0/.

The contour maps in Fig. 4 illustrate charge distributions over the butterfly-shaped structures **1–11** so that positive and negative charges, respectively, are displayed by yellow and red colors. It is seen that the most asymmetric charges distribution happens for DBT5-COMe which results in the biggest dipole moment of 8.5837 D for this sample, see Table 1. Indeed, molecules demonstrating more symmetric charge distributions have smaller dipole moments and vice versa. As a result, the DBT5-H, DBT5-CN and DBT5-CF_3_ with the lowermost dipole moments of 0.0030, 0.0028 and 0.0017 D, respectively, illuminate the most symmetric charge distributions.

### Electronic properties

**Figure 5:**
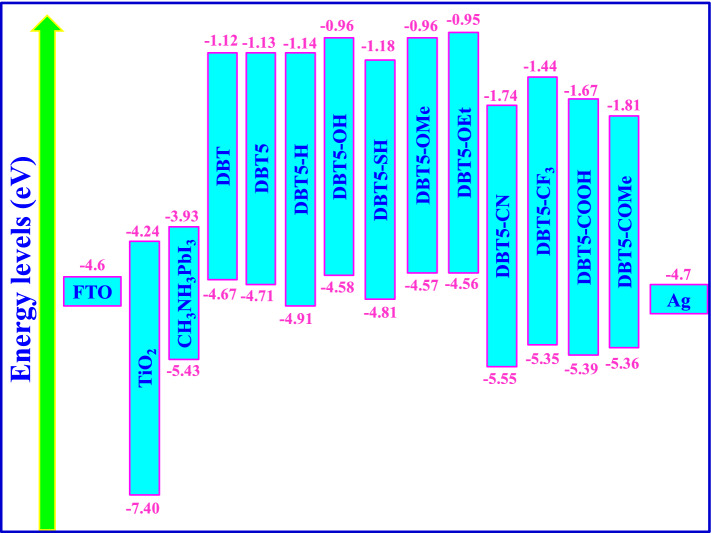
The energy levels diagrams of the FTO, TiO_2_, MAPbI_3_ perovskite, designed HTMs, and Ag cathode.

It is known that HOMO and LUMO energy levels of HTM influence its hole transfer property and in turn affect performance of solar cell. The energy levels diagrams of FTO, TiO_2_, MAPbI_3_ perovskite, samples **1–11** and Ag cathode are presented in Fig. 5. Notably, the HOMO energy of an appropriate HTM for the PSC must be positioned upper than the valence band energy of MAPbI_3_ (− 5.43 eV)^51^. As the HOMO levels of all samples except for the DBT5-CN (− 5.55 eV) are located higher than MAPbI_3_ valence band, all molecules but DBT5-CN are beneficial HTMs with appropriate energy level alignments which lead to successful hole transport from MAPbI_3_ toward the HTMs. Also, the deepest HOMO energies of − 5.39, − 5.36 and − 6.35 eV belong to the DBT5-COOH, DBT5-COMe and DBT5-CF_3_ molecules, respectively, which may result in easier hole transfer from MAPbI_3_ to the HTMs and the Ag cathode.

As the HOMO energies of DBT5-COOH, DBT5-COMe and DBT5-CF_3_ samples are deeper than HOMO level of Spiro-OMeTAD (− 5.09 eV)^47^, it can be predicted that they are superior to the Spiro-OMeTAD. The HOMO levels are − 4.671, − 4.707, − 5.38, − 4.905, − 4.61, − 4.583, − 4.810, − 4.573, − 4.555, − 5.555, − 5.350, − 5.396 and − 5.357 eV, respectively, for the DBT, DBT5, DBT5-H, DBT5-OH, DBT5-SH, DBT5-OMe, DBT5-OEt, DBT5-CN, DBT5-CF_3_, DBT5-COOH and DBT5-COMe. Evidently, the HOMO energies of DBT, DBT5-OH, DBT5-OMe and DBT5-OEt samples are upper than the Fermi level of Ag (− 4.7 eV)^59^, which reveal they cannot transfer holes toward the Ag electrode. Consequently, such compounds are not suitable HTMs for PSCs fabricated by the Ag cathode.

The LUMO energy of a favorable HTM of a PSC must be located higher than MAPbI_3_ conduction band (− 3.93 eV)^60^ in order to stop backward movement of photo-created electrons from MAPbI_3_ toward the Ag cathode. Figure 5 shows that all LUMO levels are positioned at upper energies than MAPbI_3_ conduction band confirming they are suitable materials which effectively inhibit the electron transport from perovskite to the cathode.

**Figure 6:**
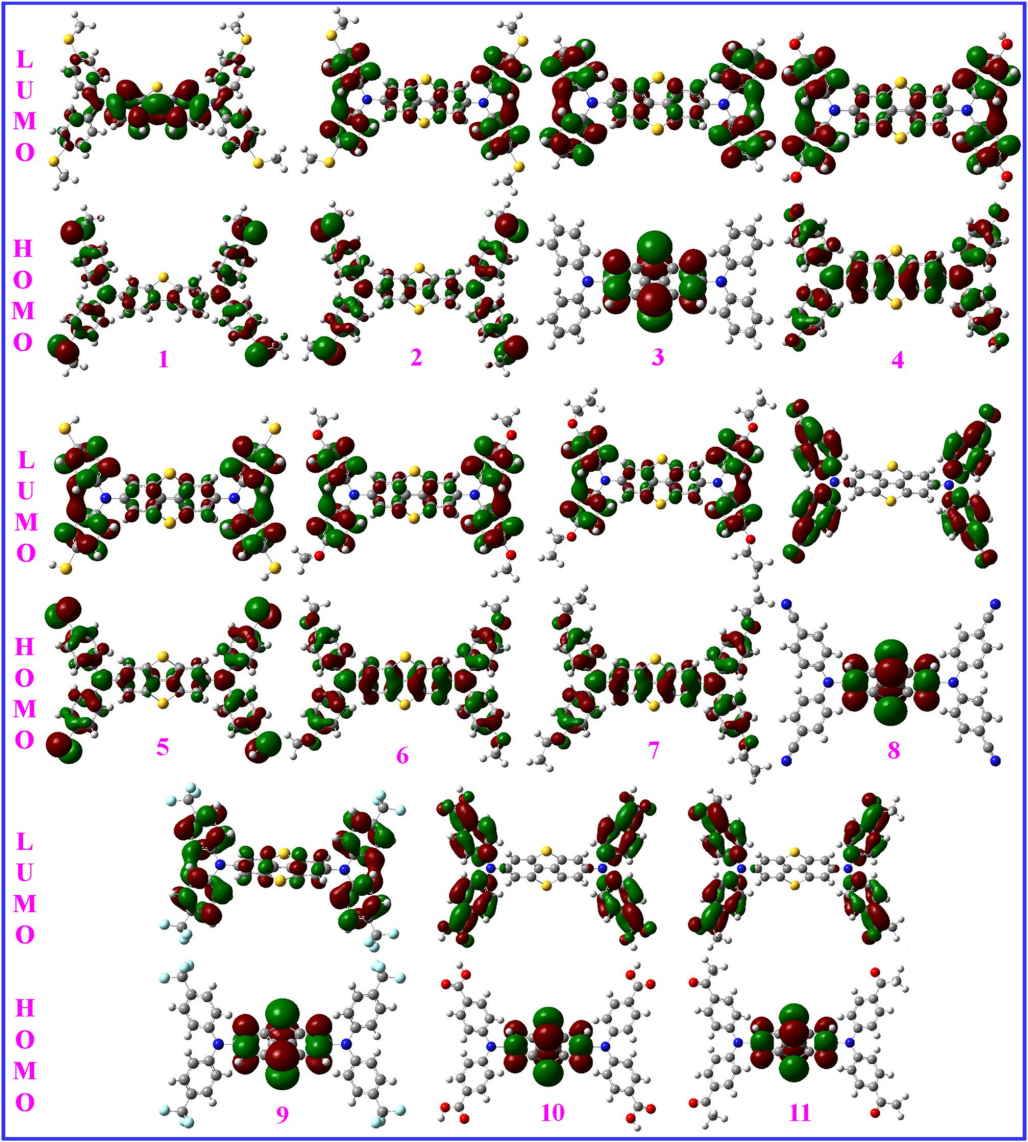
The HOMO (green) and LUMO (red) frontier molecular orbitals of compounds **1–11**. This figure was created by Gauss View 5 software which can be found at: https://gaussview.software.informer.com/5.0/.

Figure 6 presents HOMO and LUMO distributions on samples **1–11**. As can be seen, HOMO orbitals are practically positioned over the entire structures in all samples except for DBT5-H, DBT5-CN, DBT5-CF_3_, DBT5-COOH and DBT5-COMe in which the HOMO orbitals are only distributed onto the central dibenzodithiophene part. On the other hand, the LUMO orbitals are situated on the entire structures but the X substituents in all samples except for DBT5-CN, DBT5-COOH and DBT5-COMe in which the LUMO orbitals are nearly dispersed over the whole molecules but the central dibenzodithiophene fragment. It may be suggested that the molecules with higher HOMO distributions compared to the LUMO orbitals may indicate more effective hole transport properties than the electron transfer effects.

Figure S2 shows the density of states (DOS) spectra for molecules **1–11** in which the green and blue regions depict occupied and virtual orbitals. As well, there is a gap between occupied and virtual orbitals which is called bandgap (E_g_) which is HOMO–LUMO energy. The E_g_ values in Table 2 exhibit that DBT5-CF_3_ and DBT5-COMe respectively have the largest and the lowest bandgaps of 3.906 and 3.544 eV. As well, the E_g_ value enhances from 3.550 eV (in DBT) to 3.574 eV (in DBT5) signifying addition of another thiophene ring leads to increasing the E_g_. Comparing the similar DBT5-OMe, DBT5-OEt and DBT5-COOH, DBT5-COMe depicts that replacement of substituent with an electron donating group decreases the E_g_ amount.

**Table 2:** Quantum molecular descriptors for optimized structures **1–11** calculated at B3LYP-D3/6-31G(d,p) level.

HTM	E_HOMO_	E_LUMO_	E_g_	I	A	µ	χ	η	ω	IP
DBT	− 4.671	− 1.122	3.550	4.671	1.122	− 2.897	2.897	1.775	2.364	4.589
DBT5	− 4.707	− 1.132	3.574	4.707	1.132	− 2.919	2.919	1.787	2.384	4.613
DBT5-H	− 4.905	− 1.139	3.767	4.905	1.139	− 3.022	3.022	1.883	2.424	4.769
DBT5-OH	− 4.583	− 0.956	3.627	4.583	0.956	− 2.770	2.770	1.813	2.115	4.495
DBT5-SH	− 4.810	− 1.180	3.631	4.810	1.180	− 2.995	2.995	1.815	2.471	4.707
DBT5-OMe	− 4.573	− 0.961	3.612	4.573	0.961	− 2.767	2.767	1.806	2.119	4.481
DBT5-OEt	− 4.555	− 0.950	3.606	4.555	0.950	− 2.752	2.752	1.803	2.101	4.462
DBT5-CN	− 5.555	− 1.739	3.816	5.555	1.739	− 3.647	3.647	1.908	3.485	5.339
DBT5-CF_3_	− 5.350	− 1.444	3.906	5.350	1.444	− 3.397	3.397	1.953	2.955	5.141
DBT5-COOH	− 5.396	− 1.674	3.722	5.396	1.674	− 3.535	3.535	1.861	3.356	5.179
DBT5-COMe	− 5.357	− 1.813	3.544	5.357	1.813	− 3.585	3.585	1.772	3.627	5.151

I =  − E_HOMO_, A = − E_LUMO_, µ =  − (I + A)/2, χ =  − µ, η = (I–A)/2, ω = µ^2^/2η.

Diagrams of HOMO and LUMO energies against Hammett para-substituent constant (σ_p_)^61^ are provided in Fig. S3. Apparently, almost linear lines of quite high regressions values (R^2^ = 0.9271 and 0.8822) are measured for E_HOMO_ and E_LUMO_ diagrams versus Hammett constants confirming the latter is not a highly linear relationship and shows some deviation from linearity. Therefore, it may be stated that varying the para substituents substantially affects the HOMO levels whereas the LUMO energies are not greatly changes. Moreover, the non-linear diagram of LUMO against Hammett constants is due to the presence of DBT5-CF_3_. Notably, four electron withdrawing DBT5-CN, DBT5-CF_3_, DBT5-COOH and DBT5-COMe samples exhibit the most negative HOMO, LUMO levels within the ranges of − 5.350 to − 5.555 and − 1.444 to − 1.739, respectively; the neutral DBT5-H shows mediocre amounts of − 4.905 and − 2.06 eV but electron donating molecules reveal less negative HOMO, LUMO levels within the ranges of − 4.555 to − 4.905 and − 0.950 to − 1.180 eV, respectively. It should be noted that despite O–H substituent is recognized as an electron withdrawing moiety, herein it can act as an electron donating moiety as its HOMO, LUMO levels are nearly similar to the energies of electron donating groups. Therefore, it is found that the O–H group can form C=O bond (as H^+^O = C_6_H_4_^−^) with phenyl group (see Fig. S1) validating the OH substituent on aromatic phenyl ring can illustrate electron donating features.

### Molecular descriptors

To explore electronic and chemical properties of compounds **1–11**, their molecular were attained and the results are provided in Table 2. Three η = (I–A)/2, χ = (I + A)/2 and µ = – χ formula were used to obtain reactivity and structural stability, where η, µ and χ represent global hardness, chemical potential and electronegativity, respectively. Besides, I =  − E_HOMO_ and A =  − E_LUMO_, respectively, specify vertical ionization and electron affinity^62^. Usually, lower E_g_, µ and η amounts exhibit more chemical reactivity and accelerated charge transport for a material. Furthermore, an electrophilic molecule reveals a superior electrophilicity index which is estimated using the formula ω = µ^2^/2η.

It is observed in Table 2 that the DBT5-COMe shows the lowermost E_g_ = 3.544 eV plus η = 1.772 eV but a medium µ =  − 3.585 eV. On the other hand, DBT5-CF_3_ displays the utmost E_g_ = 3.906 eV and η = 1.953 eV but a moderate µ =  − 3.397 eV. Therefore, it can be proposed that the DBT5-COMe and DBT5-CF_3_ have the maximum and minimum chemical activities with perovskite and Ag cathode materials, respectively. Additionally, the DBT5-OEt with the lowest µ =  − 2.752 eV may display moderate reactivity. Moreover, the DBT5-OEt illustrates the smallest ω = 2.101 eV approving its lowest electron affinity which is beneficial for an efficient hole transport material.

The chemical stability of samples **1–11** was evaluated by comparing their hardness (η) values so that a material with a greater η exhibits a superior chemical stability. As can be seen, the η values vary as X = DBT5-CF_3_ (1.953 eV) > DBT5-CN (1.908 eV) > DBT5-H (1.883 eV) > DBT5-COOH (1.861 eV) > DBT5-SH (1.815 eV) > DBT5-OH (1.813 eV) > DBT5-OMe (1.806 eV) > DBT5-OEt (1.803 eV) > DBT5 (1.787 eV) > DBT (1.775 eV) > DBT5-COMe (1.772 eV). Accordingly, the electron withdrawing DBT5-CF_3_ shows the maximum structural stability and neutral DBT5-H exhibits more stability than other materials. Hence, the DBT5-CF_3_ with highest stability may be recognized as the most important HTM. Nonetheless, further characteristics of all compounds **1–11** must be explored to introduce the best sample.

The ionization potential (IP) values of samples **1–11** are acquired to estimate their hole injection properties because a lower IP demonstrates that the molecule has an enhanced hole injecting capacity. Comparing the IP values of all materials shows that the DBT5-CN and DBT5-OEt, respectively, have maximum and minimum IP values of 5.339 and 4.462 eV. Therefore, the DBT5-OEt is nominated as the best sample according to the IP values. The IP and I values of similar DBT5-OMe, DBT5-OEt and DBT5-COOH, DBT5-COMe samples illustrate that replacing the substituent with an electron donating group decreases the IP and I amounts. As well, all of vertical ionization (I =  − E_HOMO_) values are entirely in agreement with the IP values as DBT5-CN and DBT5-OEt exhibit the biggest and the lowermost I values of 5.555 and 4.555 eV, respectively.

To compare the electron capture properties of samples **1–11**, their vertical electron affinities (A =  − E_LUMO_) were achieved, see Table 2. As a suitable HTM for the PSC device, it should indicate the least electron affinity due to it must accept the hole from the MAPbI_3_ perovskite and transport it toward Ag cathode. The lowest and the highest electron affinities of 0.950 and 1.813 eV are attained for the DBT5-OEt and DBT5-COMe, respectively. Thus, the DBT5-OEt may be chosen as the best HTM considering the A values.

A comparison of molecular descriptors obtained for compounds **1–11** allows to offer the most promising HTM. Briefly, the DBT5-COMe shows the lowermost E_g_ = 3.544 eV plus η = 1.772 eV indicating its maximum chemical activity. The DBT5-OEt has the lowest ω = 2.101 eV which reveals it has the least electron affinity. The DBT5-CF_3_ displays the biggest η = 1.953 eV confirming its structure has the maximum stability. The DBT5-OEt illustrates smallest IP = 4.462 eV, I = 4.555 and A = 0.950 eV that are very advantageous for an effective HTM. However, as mentioned in the previous section, the DBT5-OEt cannot transfer holes to the Ag cathode as its HOMO energy is upper than Fermi level of Ag electrode. Hence, both of DBT5-COMe and DBT5-CF_3_ can be suggested as the most desirable HTMs for PSCs. It must be noted, however, that extra data must be achieved and compared for materials **1–11** to find the best HTM.

### IR, UV–Vis and PL spectra

**Figure 7:**
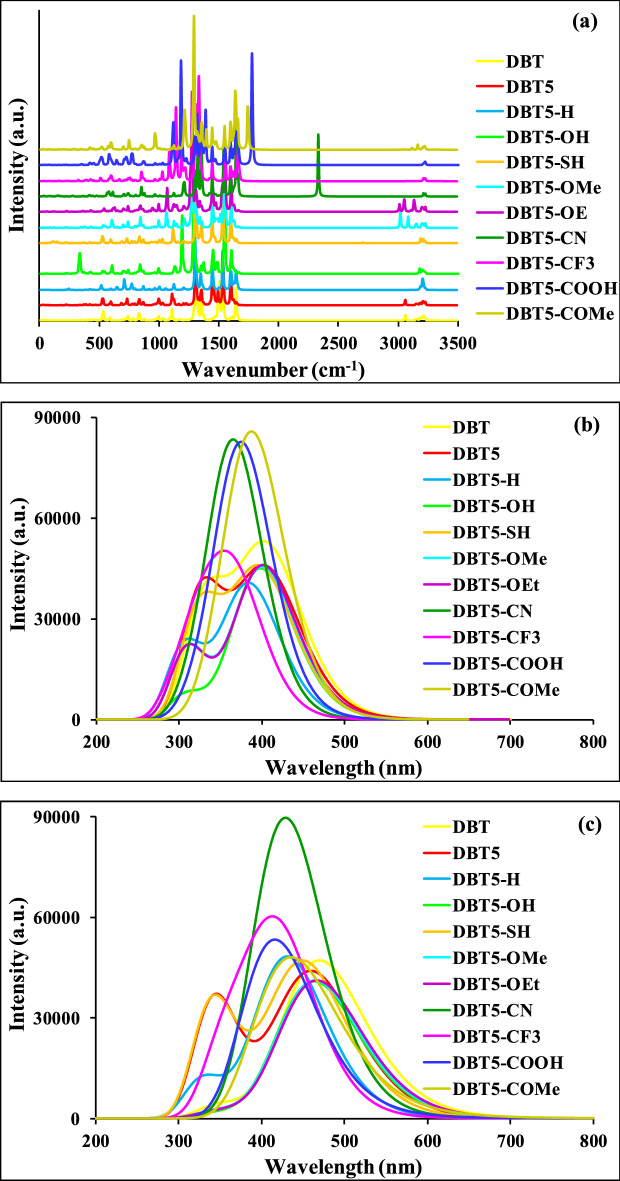
The (**a**) infrared (IR), (**b**) UV–Vis absorption and (**c**) PL emission spectra of compounds **1–11**. This figure was created by Gauss View 5 software which can be found at: https://gaussview.software.informer.com/5.0/.

The IR, UV–Vis absorption and PL emission spectra of samples **1–11** were achieved to study their functional groups and optical properties. Figure 7a demonstrates the IR spectra of compounds **1–11** which show by varying substituents, several peaks with diverse intensities are appeared. The bands at about 500–800 cm^−1^ correspond to bending of = C–H bonds. Besides, some peaks placed near 900, 1000, 1050, 1100, 1150 cm^−1^ can be attributed to vibrational stretchings of C–C, C–S, C–N, C–O, C–F bonds, respectively^63^. The bands situated at around 1350 and 1550 cm^−1^ are due to asymmetric and symmetric vibrational stretchings of C=C bonds^64^. The bands located near 3700 and 1650 cm^−1^ are correlated to vibrational stretching and bending, respectively, of O–H bonds but the peaks at about 3000 cm^−1^ are owing to stretching of C–H bonds^65^.

The UV–Vis spectra of all samples are shown in Fig. 7b and a summary of spectral data including oscillator strengths (*f*), maximum absorbance wavelengths (λ_abs_^max^), main transitions and LHEs are presented in Table 3. It is seen that all compounds exhibit two peaks within the range of about 250 to 550 nm except for four molecules DBT5-CN, DBT5-CF_3_, DBT5-COOH and DBT5-COMe containing electron withdrawing substituents which only indicate one peak. Furthermore, three DBT5-CN, DBT5-COOH and DBT5-COMe compounds exhibit the highest peak intensity and among them the DBT5-COMe displays the highest intensity.

**Table 3:** The maximum absorbance wavelength (λ_abs_^max^), oscillator strength (*f*), LHE, and the most important transitions for the HTMs **1–11.**

HTM	Label	λ_abs_^max^ (nm)	*f*	LHE	Main configuration
1	DBT	407.84	1.2263	0.9406	H → L (97.77%)
2	DBT5	406.99	1.0646	0.9138	H → L (96.82%)
3	DBT5-H	385.46	0.9791	0.8951	H → L (97.73%)
4	DBT5-OH	399.09	1.0839	0.9176	H → L (97.99%)
5	DBT5-SH	400.51	1.0638	0.9137	H → L (97.77%)
6	DBT5-OMe	401.50	1.0935	0.9194	H → L (97.93%)
7	DBT5-OEt	402.40	1.1074	0.9219	H → L (97.94%)
8	DBT5-CN	372.94	0.9789	0.8950	H → L + 2 (97.24%)
9	DBT5-CF_3_	371.50	0.9184	0.8793	H → L (97.54%)
10	DBT5-COOH	381.51	1.0038	0.9009	H → L + 2 (96.82%)
11	DBT5-COMe	390.61	1.0081	0.9018	H → L + 2 (95.94%)

*LHE* light harvesting efficiency, *H* HOMO, *L* LUMO.

Evidently, λ_abs_^max^ values are different for samples **1–11** and change in the range of 371.50 to 407.84 nm, respectively, for the DBT5-CF_3_ and DBT, respectively, so that the main transitions associated with these peaks are H → L (97.54%) and H → L (97.77%). In addition, the LHE values of all molecules are high which confirm they have great capabilities of light absorption. The LHE of similar DBT5-OMe, DBT5-OEt and DBT5-COOH, DBT5-COMe molecules elucidate that replacing the substituents with electron donating groups increases the LHE amounts. The LHE values vary from 0.8793 (for DBT5-CF_3_) to 0.9406 (for DBT) validating all of these materials are beneficial HTMs for PSCs.

Figure 7c displays the PL emission spectra of molecules **1–11** and Table 4 lists E_em_^max^, λ_em_^max^, f_em_^max^, Stokes shifts, Exciton binding energy (E_b_) and radiation lifetime (τ) of HTMs **1–11**. The PL spectra of DBT5-OH, DBT5-CN, DBT5-CF_3_, DBT5-COOH and DBT5-COMe show one broad peak whereas other molecules demonstrate two maxima or a sharp peak plus a weak shoulder while a broad peak plus a shoulder. The λ_em_^max^ values change from 406.18 nm (in DBT5-COOH) to 469.85 nm (in DBT). Additionally, all λ_em_^max^ values are greater than their associated absorption λ_abs_^max^ values. As well, greater λ_em_^max^ and λ_abs_^max^ are obtained for the DBT compared to that of DBT5 which lead to greater optical bandgaps for DBT5. A comparison of DBT5-OMe, DBT5-OEt and DBT5-COOH, DBT5-COMe similar HTMs approves that the λ_em_^max^ and λ_abs_^max^ values have red shifts by replacing the substituents with electron donating groups. These results are in consistent with the E_g_ values measured for these samples.

**Table 4:** The maximum emission wavelength (λ_em_^max^), Stokes shift, E_em_^max^, f_em_^max^, radiation lifetime (τ), and E_b_ of HTMs **1–11**.

HTM	Abbreviation	λ_em_^max^ (nm)	Stokes shift (nm)	E_em_^max^ (eV)	f_em_^max^	Τ (ns)	E_b_ (eV)
1	DBT	469.85	62.01	2.639	1.162	0.118	0.911
2	DBT5	460.28	53.29	2.694	1.081	0.121	0.881
3	DBT5-H	431.54	46.08	2.873	1.186	0.097	0.894
4	DBT5-OH	463.73	64.64	2.674	1.008	0.132	0.953
5	DBT5-SH	450.68	50.17	2.751	1.153	0.109	0.880
6	DBT5-OMe	465.09	63.59	2.666	1.014	0.132	0.947
7	DBT5-OEt	467.04	64.64	2.655	1.012	0.134	0.951
8	DBT5-CN	435.58	62.64	2.846	1.511	0.078	0.970
9	DBT5-CF_3_	421.45	49.95	2.942	1.347	0.082	0.964
10	DBT5-COOH	406.18	24.67	3.052	0.992	0.103	1.465
11	DBT5-COMe	421.66	31.05	2.190	0.833	0.238	1.354

Stokes shift exhibits the wavelengths difference of the UV–Vis and PL peaks. Hence, Stokes shift is larger when PL peak shows a greater red shift toward visible spectral region. The Stokes shift corresponds to losing of energy by the absorbed photons via a non-radiative mechanism that results in decreasing average energy of emitted photons. Notably, the radiative PL emission is correlated to the recombination of holes with electrons that is unfavorable in photovoltaics. Thus, superior Stokes shifts upon non-radiative PL emissions are more advantageous for PSC devices. As can be seen, the lowest Stokes shift of 24.67 nm is achieved for DBT5-COOH while the highest value of 64.64 nm is attained for both DBT5-OH and DBT5-OEt samples. Also, adding another thiophene ring to the DBT molecule decreases the Stokes shift from 62.01 in DBT to 53.29 nm in DBT5. Moreover, the Stokes shifts increase by replacement of X substituent with electron donating moieties in DBT5-OMe, DBT5-OEt as well as DBT5-COOH, DBT5-COMe.

The radiation lifetime (τ) values were provided to estimate lifetimes of radiative recombination between holes and electrons, i.e. greater amounts exhibit longer recombination processes but smaller values illustrate shorter recombination. Table 4 displays that the shortest and longest τ values of 0.078 and 0.238 ns belong to the DBT5-CN and DBT5-COMe samples, respectively, confirming the former is the most encouraging sample for a PSC device. Comparing similar DBT5-OMe, DBT5-OEt and DBT5-COOH, DBT5-COMe HTMs with τ values of 0.132, 0.134 and 0.103, 0.238 ns illuminates that radiation lifetime enhances by replacement of X substituent with a more electron donating group. Also, the DBT5-H (0.097 ns) with the neutral substituent reveals a smaller radiation lifetime than those of the DBT (0.118 ns) and DBT5 (0.121 ns). Notably, the DBT5-CF_3_ including electron withdrawing groups shows a small τ = 0.082 ns indicating its favorable short electron–hole recombination that is highly suitable to improve the PSC efficacy.

The binding energies of Excitons (electron–hole couples) are measured and showed by E_b_ values in Table 4. The E_b_ amount corresponds to the PSC performance, i.e. a higher E_b_ illustrates stronger Coulombic attraction of electron and hole that causes difficult separation of Exciton binding, less current density and lower PSC efficiency. Noticeably, the lowest and the largest E_b_ values of 0.880 and 1.465 eV are obtained for the DBT5-SH and DBT5-COOH, respectively. In addition, the DBT5 and DBT5-H depict low E_b_ values which confirm they can lead to satisfactory results for the PSCs assembled using these materials. Notably, substituent replacement by electron withdrawing groups in DBT5-COOH as well as DBT5-COMe enhances the E_b_ amount. As a result, Exciton binding separation of happens the easiest in DBT5-SH which may afford the utmost current density for the PSCs.

### Hole transport properties

**Figure 8:**
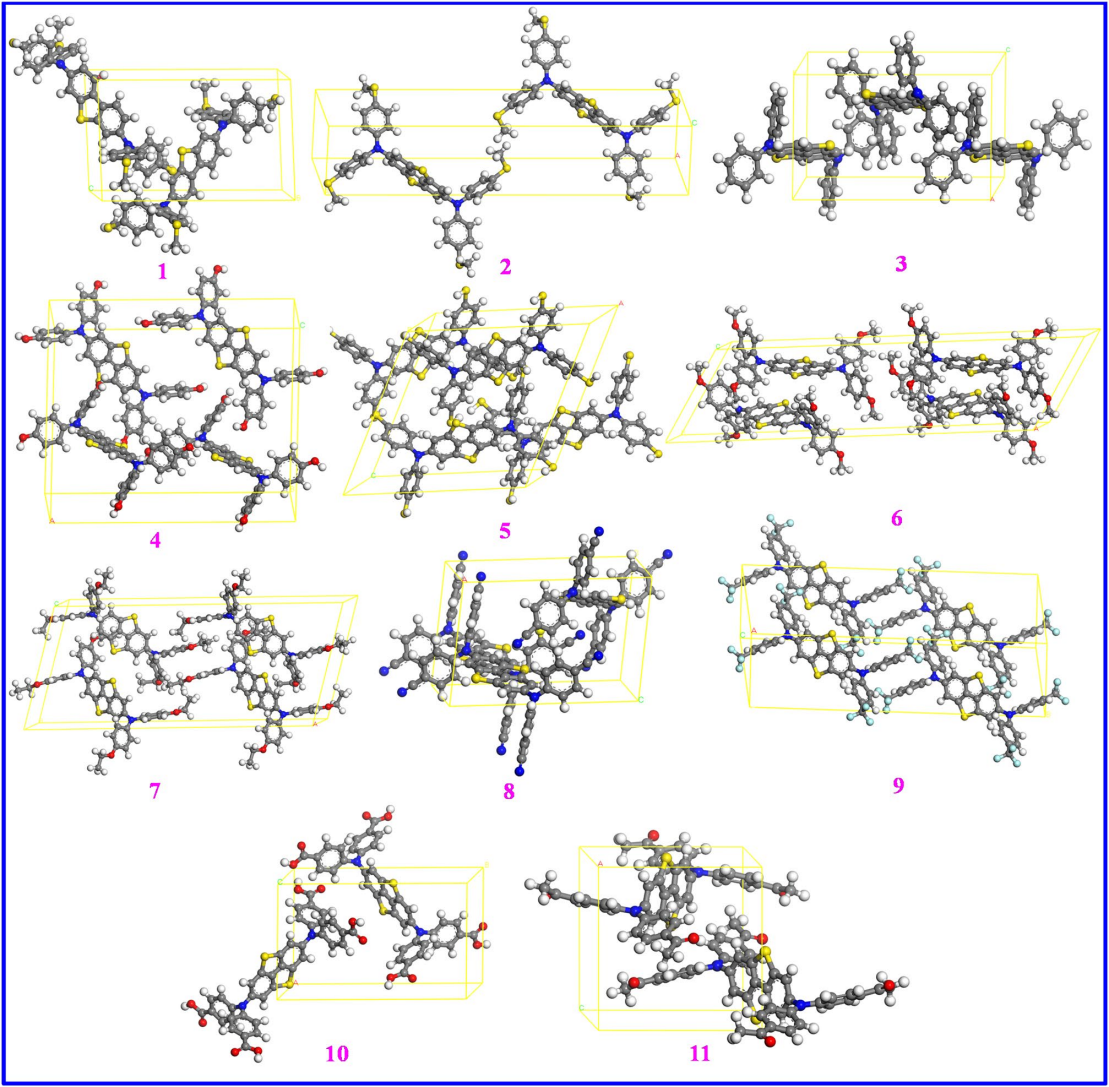
The most stable unit cells of the crystalline structures of the designed HTMs. This figure was created by Accelrys, Materials studio software which can be found at: https://www.3ds.com/products-services/biovia/products/molecular-modeling-simulation/biovia-materials-studio/.

The hole mobility of a material has a great influence onto PSC performances as it changes both the V_OC_ and current density of photovoltaic devices. The hole mobility data of HTMs were achieved by computing ten crystalline structures for each sample to obtain the most stable (lowest total energy) molecule that was selected to calculate the hole mobility. Figure 8 illustrates the most stable predicted unit cells for crystalline structures of all samples. Besides, crystallographic data for the most stable crystal structures of samples **1–11** are provided in Table 5. The dimer structures of compounds **1–11** with the DBT5 core were used to estimate their hole hopping characteristics (Fig. S4) that demonstrate end-to-end configuration for all samples but the face-to-face configuration for **6**. It is notable that in face-to-face arrangement, the π–π stacking intermolecular interactions are able to boost electronic couplings.

**Table 5:** The crystallographic data of the most stable crystalline system for the samples **1–11**.

No.	Label	Space group	Length a (Ǻ)	Length b (Ǻ)	Length c (Ǻ)	Angle α (°)	Angle β (°)	Angle γ (°)
1	DBT	P21	11.19447	17.17809	10.24107	90	67.61	90
2	DBT5	P-1	6.883414	35.31484	7.752253	94.08	88.96	76.55
3	DBT5-H	P21	9.725512	15.43115	9.448013	90	105.99	90
4	DBT5-OH	C2	24.66871	5.246973	23.45199	90	87.21	90
5	DBT5-SH	C2	18.33881	11.43984	17.63216	90	116.28	90
6	DBT5-OMe	C2	41.55289	8.581109	11.94310	90	58.04	90
7	DBT5-OEt	C2	36.56015	7.595204	15.78342	90	73.45	90
8	DBT5-CN	P21	15.62977	10.12383	10.33301	90	83.64	90
9	DBT5-CF_3_	P-1	7.87385	31.95065	11.01654	88.76	40.96	89.11
10	DBT5-COOH	P21	11.00563	18.18475	8.939933	90	76.05	90
11	DBT5-COMe	P21	11.48206	11.55336	14.11468	90	83.36	90

It should be reminded that the HOMO and LUMO energy levels in Fig. 5 showed that the five molecules including DBT, DBT5-OH, DBT5-OMe, DBT5-OEt and DBT5-CN do not have suitable band alignments and cannot transfer holes from the MAPBI_3_ to the Ag cathode. However, we calculated the hole mobility data for all samples **1–11**. The hole mobility (μ_h_), hole reorganization energy (λ_h_), hole mobility rate (k_h_), electron coupling (V), and centroid–centroid distance (r) of samples with favorable and unfavorable band alignments are given in Table 6 and Table S1, respectively.

**Table 6:** The centroid to centroid distance (r), transfer integral (V), reorganization energy (λ_h_), charge hopping rate (k_h_) and hole mobility (μ_h_) of HTM samples.

Label	r (Å)	|V| (eV)	λ_h_ (eV)	k_h_ (s^−1^)	μ_h_ (cm^2^/V/s)
DBT5	17.871	0.00041	0.1403	1.94 × 10^9^	3.984 × 10^–4^
DBT5-H	9.635	0.00770	0.2437	1.91 × 10^11^	1.140 × 10^–2^
DBT5-SH	10.807	0.00963	0.1624	8.01 × 10^11^	6.031 × 10^–2^
DBT5-CF_3_	16.391	0.00175	0.3666	2.45 × 10^9^	4.234 × 10^–4^
DBT5-COOH	14.687	0.00084	0.3729	5.26 × 10^8^	7.311 × 10^–5^
DBT5-COMe	11.650	0.00526	0.3495	2.67 × 10^10^	2.336 × 10^–3^

Among samples with favorable band alignments listed in Table 6, the biggest and smallest λ_h_ values of 0.1403 and 0.3729 eV, respectively, are measured for DBT5 and DBT5-COOH. Besides, k_h_ and μ_h_ exhibit alike changes by altering the HTMs and decrease in the order of DBT5-SH > DBT5-H > DBT5-COMe > DBT5-CF_3_ > DBT5 > DBT5-COOH. Noticeably, the samples composed of SH and H substituents show the greatest μ_h_ of 6.031 × 10^–2^ and 1.140 × 10^–2^ cm^2^/V/s whereas other molecules exhibit smaller k_h_ and μ_h_ amounts. Notably, the hole mobility achieved for the DBT (7.805 × 10^–2^ cm^2^/V/s) is greater than the value measured for the champion DBT5-SH material but it is observed in Fig. 5 that the DBT does not display a suitable band alignment with respect to the Ag cathode electrode. In addition, the μ_h_ values of all compounds except for the DBT5-COOH in Table 6 are greater than that of the reference DBT5 molecule containing the SMe substituent. Comparing the two similar DBT5-COMe and DBT5-COOH materials exhibits that the μ_h_ value is almost 100 times greater for the latter. The calculated and experimental μ_h_ values of Spiro-OMeTAD, respectively, are 5.65 × 10^–3^ and 4.53 × 10^–4^ cm^2^/V/s^47^. When the μ_h_ data of the DBT5-SH and DBT5-H are compared with those measured for the Spiro-OMeTAD, it is found that the hole mobilities of DBT5-SH and DBT5-H are about 10 and 100 times superior to the calculated and experimental μ_h_ values for the Spiro-OMeTAD. This can be due to the high conjugations of π electrons in this compound which boost its hole mobility. As the PSCs assembled by the DBT HTM illustrated a high PCE = 21.12% and fill factor = 83.25%^34^, it can be assumed that the PSCs with the DBT5-SH as HTM will illuminate high PCEs analogous to or larger compared to the PCEs of devices containing Spiro-OMeTAD. Also, the two DBT5-H and DBT5-COMe samples are expected to indicate high performance for PSCs as they have large hole mobilities.

### Performances of PSCs

The performance of a PSC is measured using the formula PCE = $\frac{{\mathrm{J}}_{\mathrm{sc}} {\mathrm{V}}_{\mathrm{oc}}\mathrm{ FF}}{{\mathrm{P}}_{\mathrm{in}}}$^66^, where FF and P_in_ show the fill factor and incident power (100 mW/cm^2^) while V_OC_ and J_SC_ respectively exhibit the open circuit voltage and short circuit current density. The PCE greatly boosts by enhancement of both V_OC_ and J_SC_ which are the highest voltage measured at zero current density and maximum current density at zero voltage, respectively. To estimate the PCEs of PSCs based on HTM samples with suitable band alignments, the experimentally reported J_SC_ = 22.7 mA/cm^2^^34^ was used and the V_OC_ values were estimated using formula VOC = ELUMO of acceptor − EHOMO of donor − 0.3/*e*^67^, in which MAPbI_3_ and HTM are electron acceptor and donor materials, *e* stands for the unit electronic charge and 0.3 shows a constant signifying voltage decrease. The conduction band of MAPbI_3_ (− 3.93 eV)^60^ and the HOMO energies of HTMs were utilized to estimate V_OC_ values. Hence, a HTM indicating a deeper HOMO level can produce a larger V_OC_.

Table 7 and Table S2 demonstrates the photovoltaic performance parameters for HTM samples. It is found that the DBT5-COOH affords the maximum V_OC_ = 1.166 eV confirming it can be the most effective HTM for the PSCs. Additionally, the V_OC_ enhances from 0.476 eV (in DBT5) to higher values in other HTMs indicating substitution of SMe groups by other groups increases the V_OC_ value. As well, the neutral H substituent in DBT5-H affords a greater V_OC_ (0.675 eV) than those measured for the DBT5 and DBT5-SH HTMs. Three HTMs including DBT5-COOH, DBT5-COMe and DBT5-CF_3_ exhibit highest V_OC_ amounts of 1.166, 1.127 and 1.120 eV, respectively, which verify these materials have a high capacity for application in PSCs. Accordingly, the DBT5-COOH with the utmost V_OC_ is the most promising material for the PSC device fabrication.

**Table 7:** The photovoltaic performance parameters for HTM samples.

HTM	V_OC_ (eV)	FF	PCE (%)
DBT5	0.476	0.798	8.627
DBT5-H	0.675	0.843	12.913
DBT5-SH	0.580	0.824	10.854
DBT5-CF_3_	1.120	0.893	22.694
DBT5-COOH	1.166	0.896	23.707
DBT5-COMe	1.127	0.893	22.857

The FF values are attained using the formula FF = $\frac{\frac{\mathrm{e }{\mathrm{V}}_{\mathrm{OC}}}{{\mathrm{K}}_{\mathrm{B}}\mathrm{ T}} -\mathrm{ ln}\left(\frac{\mathrm{e }{\mathrm{ V}}_{\mathrm{OC}}}{{\mathrm{K}}_{\mathrm{B}}\mathrm{ T}} + 0.72\right)}{\frac{\mathrm{e}{\mathrm{ V}}_{\mathrm{OC}}}{{\mathrm{K}}_{\mathrm{B}}\mathrm{ T}}+1}$^68^, where *e* is the unit electronic charge, K_B_ = 8.61733034 is the Boltzmann constant and the temperature equals T = 298 K. Obviously, the largest FF and PCE values of 0.896 and 23.707%, respectively, are achieved for DBT5-COOH. Furthermore, all of the V_OC_, FF and PCE values change in the order of DBT5 < DBT5-SH < DBT5-H < DBT5-CF_3_ < DBT5-COMe < DBT5-COOH (except for the equal FF = 0.893 measured for the DBT5-CF_3_ and DBT5-COMe). All photovoltaic parameters of three samples including DBT5-CF_3_, DBT5-COMe and DBT5-COOH illustrate significant differences with those of other HTMs. This result verifies that presence of electron withdrawing CF_3_, COMe and particularly the COOH group on the *para*-position of phenyl rings extremely boosts the PSCs performances.

## Conclusion

The structural, optical, electronic and hole transfer features of some butterfly-shaped HTMs based on dibenzo[b,d]thiophene (DBT) and dibenzo-dithiophene (DBT5) cores were explored using DFT computations. The lowest and highest ΔE_solvation_ values varied from − 5.185 to − 18.140 kcal/mol for the DBT5-H and DBT5-CN, respectively, and the high ΔE_solvation_ values of − 18.140, − 15.929, − 14.756 and − 13.460 kcal/mol for the DBT5-CN, DBT5-COOH, DBT5-COMe and DBT5-OH HTMs confirmed their high solubility and stability. The HTMs with properly aligned LUMO and HOMO energy levels with respect to those of MAPbI_3_ perovskite and Ag cathode could successfully inject holes. Three HTMs including DBT5-COOH, DBT5-COMe and DBT5-CF_3_ exhibited highest V_OC_ amounts of 0.856, 0.817 and 0.810 eV, respectively. Almost linear lines of rather great regressions (R^2^ = 0.9271 and 0.8822) were measured for E_HOMO_ and E_LUMO_ diagrams versus Hammett constants approving the latter was not a highly linear relationship and showed some deviation from linearity. Molecular descriptors established that the DBT5-COMe had the lowermost E_g_ = 3.544 eV and η = 1.772 eV indicating its maximum chemical activity and DBT5-CF_3_ displayed the biggest η = 1.953 eV confirming its utmost stability. The lowest and the largest E_b_ values of 0.880 and 1.465 eV were obtained for the DBT5-SH and DBT5-COOH, respectively. The shortest and longest τ values of 0.078 and 0.238 ns belonged to the DBT5-CN and DBT5-COMe samples, respectively, confirming the former was the most encouraging sample for a PSC device. The DBT5-SH and DBT5-H exhibited the greatest μ_h_ values of 6.031 × 10^–2^ and 1.140 × 10^–2^ cm^2^/V/s which were greater than that of the reference DBT5 molecule (3.984 × 10^–4^ cm^2^/V/s) and about 10 and 100 times superior to the calculated and experimental μ_h_ values for the famous Spiro-OMeTAD. The largest V_OC_, FF and PCE values of 1.166 eV, 0.896 and 23.707%, respectively, were achieved for DBT5-COOH which evidenced it was the most promising material for the PSCs fabrication.

## Supplementary Information

Supplementary Information.

## Data Availability

The computational data will be delivered on reasonable request. If someone wants to request the data from this study, please contact Zahra Shariatinia (shariati@aut.ac.ir).
