# Supplementary material for: Molecular engineering of several butterfly-shaped hole transport materials containing dibenzo[b,d]thiophene core for perovskite photovoltaics
Source: Sci Rep. 2022 Aug 17;12:13954. doi: 10.1038/s41598-022-18469-1 (PMC9386032; doi:10.1038/s41598-022-18469-1)
Supplement: Supplementary file 1 — Supplementary Information. [file 41598_2022_18469_MOESM1_ESM.docx]

**Molecular engineering of several butterfly-shaped hole transport materials containing dibenzo[*b,d*]thiophene core for perovskite photovoltaics**

**Zahra Shariatinia^1,🖂^, Seyed-Iman Sarmalek^1^**

^1^Department of Chemistry, Amirkabir University of Technology (Tehran Polytechnic),

P.O.Box:15875-4413, Tehran, Iran. **^🖂^**Correspondence to: email: [shariati@aut.ac.ir](mailto:shariati@aut.ac.ir)

**Table S1.**

The centroid to centroid distance (r), transfer integral (V), reorganization energy (λ_h_),

charge hopping rate (k_h_) and hole mobility (μ_h_) of samples with unfavorable band alignments.

| HTM | r (Å) | \|V\| (eV) | λ_h_ (eV) | k_h_ (s^-1^) | μ_h_ (cm^2^V^-1^s^-1^) |
| --- | --- | --- | --- | --- | --- |
| DBT | 10.241 | 0.00909 | 0.1259 | 1.15×10^12^ | 7.805×10^-2^ |
| DBT5-OH | 17.312 | 0.00031 | 0.1560 | 9.01×10^8^ | 1.741×10^-4^ |
| DBT5-OMe | 7.281 | 0.00056 | 0.1575 | 2.88×10^9^ | 9.851×10^-5^ |
| DBT5-OEt | 18.071 | 0.00174 | 0.1571 | 2.80×10^10^ | 5.893×10^-3^ |
| DBT5-CN | 10.124 | 0.00339 | 0.3716 | 8.69×10^9^ | 5.740×10^-4^ |

**Table S2.**

The photovoltaic performance parameters for HTM samples.

| HTM | V_OC_ (eV) | FF | PCE (%) |
| --- | --- | --- | --- |
| DBT | 0.441 | 0.786 | 7.879 |
| DBT5-OH | 0.353 | 0.751 | 6.017 |
| DBT5-OMe | 0.343 | 0.746 | 5.807 |
| DBT5-OEt | 0.325 | 0.737 | 5.440 |
| DBT5-CN | 1.325 | 0.906 | 27.241 |

**Fig. S1.** The C=O double bond formation between phenyl ring and OH group

which acts as an electron donating moiety.

**Fig. S2.** The density of states (DOS) spectra for compounds **1-11**.

**Fig. S3.** Plots of the HOMO and LUMO energy levels versus the substituents constants (σ_p_).

**Fig. S4.** The dimer structures of all HTMs **1-11** used to estimate the hole mobility.
